# The Relationship Between the Development of General Offending and Intimate Partner Violence Perpetration in Young Adulthood

**DOI:** 10.1177/0886260520922340

**Published:** 2020-05-27

**Authors:** Janna Verbruggen, Christopher D. Maxwell, Amanda L. Robinson

**Affiliations:** 1 VU University Amsterdam, The Netherlands; 2 Michigan State University, East Lansing, USA; 3 Cardiff University, UK

**Keywords:** intimate partner violence, offending trajectories, general offending, young adulthood, life-course, longitudinal

## Abstract

This study examined how patterns in general offending relate to the occurrence of and likelihood of persistence in intimate partner violence (IPV) perpetration in young adulthood. The study used longitudinal data from the cohort of 18 year olds from the Project on Human Development in Chicago Neighborhoods study. Self-reported offending was measured in all three waves, and data on IPV were collected in Waves 1 and 3. Group-based trajectory modeling identified three distinct general offending trajectory groups: non-offenders, low-rate offenders, and high-rate offenders. The majority of respondents engaged in psychological IPV perpetration, and half of all young adults reported physical IPV, but prevalence rates decreased over the waves. Binary logistic regression analyses showed that those involved in offending, especially those who showed a diverse offending pattern, were at increased risk of perpetrating psychological and (severe) physical IPV, as well as to show persistence in the different forms of IPV perpetration. The findings highlight an important overlap between general crime and IPV perpetration. In recognition that IPV is often part of a broader pattern of antisocial behavior, interventions should focus on interrupting the criminal careers of *all* young offenders to reduce the prevalence and harms of IPV.

## Introduction

Life-course criminological research has shown that general offending peaks between the late teenage years and early adulthood, and decreases thereafter (Farrington et al., 2008; Gottfredson & Hirschi, 1990; Sweeten et al., 2013). Although most offenders desist from crime at some point, it is also well documented that those who continue their offending behavior into adulthood are often characterized by problematic backgrounds, and engage in serious and frequent criminal behavior (Moffitt, 1993). However, theory and research on the development of offending has largely focused on general offending or “street” crime. Specific and less visible crime types, such as intimate partner violence (IPV), are rarely included in models of general offending (Piquero et al., 2014). This gap is in part because research about IPV perpetration has often used cross-sectional designs, and the relatively small body of research taking a longitudinal approach to study IPV perpetration has developed largely separate from life-course criminological research on general offending. As a result, there is limited theoretical integration and little empirical evidence about the relationship between the development of general offending and the development of IPV perpetration during young adulthood (cf. W. L. Johnson et al., 2015). This study aims to address this gap in the literature by examining how patterns in general offending relate to the occurrence of and likelihood of persistence in IPV perpetration.

## Theoretical Framework

Life-course criminological theory argues that there is both change and stability in antisocial and criminal behavior over time (Sampson & Laub, 1993). On one hand, life-course criminological research has demonstrated that a large proportion of offenders desist from offending in young adulthood (Piquero et al., 2007; Sampson & Laub, 1993). Some argue the decrease in offending behavior is due to maturation or people “aging out” of crime (Massoglia & Uggen, 2010; Moffitt, 1993), while others assume that the social bonds that people tend to form in young adulthood promote a more conventional lifestyle (Sampson & Laub, 1993). On the other hand, research has also found considerable continuity in aggressive and antisocial behavior over time (Huesmann et al., 2009; Piquero et al., 2012). This stability in offending over the life-course is argued to be due to stable underlying characteristics (Gottfredson & Hirschi, 1990), an interaction between early individual characteristics and environmental risk factors (Moffitt, 1993), or a process of cumulative disadvantage, in which the consequences of earlier antisocial behavior reduce people’s chances of leaving their criminal behavior behind (Sampson & Laub, 1997).

In addition, general theories of crime and violence assert that certain individuals, due to a stable underlying antisocial propensity, start their criminal career early in life and are likely to engage in persistent antisocial behavior over the life-course and exhibit different forms of antisocial behavior across different social contexts, including in the context of an intimate relationship (Gottfredson & Hirschi, 1990; Moffitt, 1993). Whereas the formation of stable relationships in young adulthood is found to be an important desistance factor for general offending (Sampson & Laub, 1993), this may not be the case for the more serious offenders, for whom entering a relationship may provide another opportunity to display antisocial and violent behavior (Moffitt, 1993). Moreover, others have argued that those who engage in IPV may be more similar to, rather than different from, other violent offenders, and that general crime and violence and IPV perpetration to a significant extent have a shared etiology (Fagan & Wexler, 1987; Felson & Lane, 2010; Moffitt et al., 2000).

Therefore, following these perspectives, a considerable overlap between general offending and IPV perpetration is expected, in that those who develop a pattern of criminal behavior are also at increased risk of engaging in more frequent IPV perpetration. However, limited research explores this relationship between developmental patterns in general offending and the development of IPV perpetration. This is in part because early IPV focused theorists developed their models largely separate from theories of general offending. To illustrate, theories that scholars have developed specifically to understand IPV perpetration focus on the role of gender inequality and gender roles defined by patriarchal society (R. E. Dobash & Dobash, 1979); on characteristics of the family context, including power, conflict, and stress (Straus et al., 1980); or on the link between early experiences of family violence and child abuse and later IPV (Ehrensaft et al., 2003; Mihalic & Elliott, 1997; Widom, 1989). These theories do not consider the role that other criminal behavior plays when studying IPV perpetration, and therefore have not placed the development of IPV perpetration into a broader pattern of criminal behavior.

In contrast to the early IPV theorists, other scholars focusing on identifying personality characteristics of IPV perpetrators have considered the role of aggression and crime more broadly, revealing that those perpetrators who are violent both within and outside the family context tend to be the more serious IPV perpetrators (Holtzworth-Munroe & Stuart, 1994). Furthermore, other more recent integrated theoretical models also explicitly consider an individual’s background of antisocial behavior, alongside a variety of other factors, to explain IPV. In particular, the dynamic developmental system model considers individual risk or background factors of both partners, including antisocial behavior, when explaining IPV (Capaldi & Clark, 1998; Capaldi et al., 2005; Capaldi & Kim, 2007). However, this model focuses particularly on the relationship context, and argues that factors including interaction patterns, as well as other proximal factors such as substance use, are important for understanding the occurrence, severity, and duration of IPV perpetration.

## Prior Research

Prior research that has examined general antisocial or criminal behavior in relation to IPV perpetration provides a compelling evidence base demonstrating a considerable relationship between the two. To begin with, review studies point to general antisocial or delinquent behavior in the teenage years as an important risk factor for involvement in IPV in young adulthood (Capaldi et al., 2012; Costa et al., 2015).

Furthermore, research has shown that those involved in serious and/or persistent antisocial or criminal behavior are at an especially increased risk of perpetrating IPV. For example, using a subsample (*N* = 495) from the Christchurch Health and Development Study of a birth cohort, Woodward et al. (2002) measured antisocial behavior from ages 8 to 21, and found that young people who showed early-onset persistent antisocial behavior were at higher risk of engaging in IPV, as well as reported higher levels of IPV perpetration at age 21, compared with adolescent-onset and non-offender groups. Similarly, Moffitt et al. (2002) followed a birth cohort until age 26 as part of the Dunedin Multidisciplinary Health and Development Study. Focusing on the males (*N* = 477), they demonstrated that those with a persistent pattern of serious antisocial behavior throughout childhood and adolescence were more likely to have engaged in physical IPV at age 26, and to have a court conviction for violence against women, compared with those who showed normative antisocial behavior. Moreover, focusing specifically on violent offending, Herrenkohl et al. (2007) examined the association between patterns in violence during adolescence (ages 13–18) and IPV perpetration at age 24 using a school-based sample (*N* = 644). Using group-based trajectory modeling, they identified four violent trajectory groups. Those in the chronic violence and the late-increaser groups were more likely to report IPV in young adulthood compared with the non-offender and desister groups, although the effects became marginally significant when other proximal risk factors were taken into account. Furthermore, Piquero et al. (2014) also used group-based trajectory modeling to distinguish between different offender groups among males followed in the Cambridge Study in Delinquent Development, and found that the two groups who engaged in chronic offending over the life-course, measured from ages 10 to 40, were at increased risk of engaging in physical IPV perpetration beyond young adulthood (at age 32 and/or 48). Finally, research using officially registered rather than self-report data also indicated that a large proportion of arrested adult IPV perpetrators have a history of general offending (Buzawa & Hirschel, 2008; Hilton & Eke, 2016; Klein & Tobin, 2008; Piquero et al., 2006), and that high-rate general offenders are also likely to show high-rate IPV perpetration (Richards et al., 2013).

In addition, there are a few studies that examine the role of general antisocial behavior in relation to the likelihood of persistence in IPV perpetration. Although there is a wealth of longitudinal research on patterns of desistance and persistence in general criminal behavior, longitudinal analysis of the development of IPV perpetration is still an emerging area of work (Walker et al., 2013). Some longitudinal studies following community samples suggest that rates of physical IPV perpetration decrease throughout young adulthood. For instance, following a sample of at-risk young adults (*N* = 194) over a period of 10 years, Kim et al. (2008) found a significant decrease in physical aggression over time. Similarly, studying IPV perpetration from ages 13 to 28 in a sample of about 1,200 males and females, W. L. Johnson et al. (2015) demonstrated that the development of IPV perpetration follows a similar pattern compared with the typical age-crime curve found for general crime, although IPV perpetration peaks slightly later, around age 20, and then declines. However, research also points to stability in IPV perpetration over time in school-based (Greenman & Matsuda, 2016), and at-risk samples followed up until approximately age 30 (Shortt et al., 2012). Moreover, studies examining married and cohabitating couples indicate that stability in IPV is more likely among those who engaged in severe IPV (Caetano et al., 2005; Quigley & Leonard, 1996).

A few studies that focused on cohabitating and married couples have considered general antisocial or offending behavior in relation to the likelihood of persistence in IPV, and indicated that those involved in general antisocial or criminal behavior tended to be the more serious IPV perpetrators, and appeared more likely to persist in IPV. To illustrate, Lorber and O’Leary (2004) examined physical IPV in early marriage among 94 couples and found that the husband’s general aggression was associated with persistence in (severe) IPV perpetration. Moreover, following a sample of about 100 men to conduct a longitudinal test of Holtzworth-Munroe and Stuart’s (1994) typology, which found different types of IPV perpetrators, showed that the subgroup of generally violent/antisocial men, who behaved violently and antisocially both within and outside the home, engaged in the highest levels of IPV over the 3-year follow-up period, and were least likely to desist, compared with the other groups (Holtzworth-Munroe et al., 2003).

Finally, compared with physical IPV, psychological IPV has received less attention in the literature. However, it is important to consider psychological IPV, as research indicates that psychological IPV is both a correlate and a precursor for physical IPV (Schumacher & Leonard, 2005). Research on community samples found higher rates of psychological IPV compared with physical IPV, as well as evidence for stability over time in psychological IPV (Capaldi et al., 2003; Fritz & Slep, 2009; Kim et al., 2008; Shortt et al., 2012). Although research on the association between general crime and psychological IPV is limited, there is some evidence that involvement in antisocial behavior is related to an increased likelihood of psychological IPV perpetration (Kim et al., 2008; Magdol et al., 1998).

## The Current Study

The existing research indicates that general offending is to some extent related to IPV perpetration, and that especially persistent general offenders are at increased risk of perpetrating IPV. Moreover, those IPV perpetrators who are also involved in offending appear more likely to show persistence in IPV. However, the body of literature on this topic is comparatively small. Moreover, although general crime is a risk factor for IPV perpetration (Capaldi et al., 2012; Costa et al., 2015), relatively little is known about how differences in developmental patterns of general offending behavior are related to IPV perpetration. Furthermore, in addition to physical IPV, this study includes psychological IPV which is a form of IPV less often studied in relation to general offending. Finally, persistence and desistance in IPV perpetration over time is a research area that is only relatively recently receiving more attention (Walker et al., 2013). Thus, this study aims to add to the body of literature in this area by examining how different developmental patterns in general offending are related to different forms of IPV as well as to persistence in different forms of IPV.

Furthermore, because existing research has often used school-based samples who have been followed up to young adulthood (W. L. Johnson et al., 2015; Kim et al., 2008; Shortt et al., 2012), individuals who are cohabitating or married (Caetano et al., 2005; Lorber & O’Leary, 2004), or samples of arrested IPV perpetrators (Piquero et al., 2006; Richards et al., 2013), it is unclear to what extent findings from existing studies are comparable and generalizable. To address these sampling shortcomings, this study uses data from the Project on Human Development in Chicago Neighborhoods (PHDCN) study. This rigorous study enables us to examine the relationship between general offending and IPV perpetration in an ethnically diverse sample of urban young adults who are representative of their peers living in Chicago, from different socioeconomic backgrounds, and including the three largest race/ethnic groups in the United States, namely Caucasian, African American, and Hispanic (Sampson, 2012). Therefore, the sample captures young people from a wide range of backgrounds, including those youths whose lives are likely affected most by crime and violence.

Thus, this study examines how patterns in general offending are related to the occurrence and likelihood of persistence in IPV perpetration in a sample of urban, ethnically diverse young adults. To identify developmental patterns in general offending, we use a similar approach used in prior research in this area (Herrenkohl et al., 2007; Piquero et al., 2014). More specifically, we use group-based trajectory modeling (Nagin, 1999) to identify distinct offending pathways in the sample and then we examine whether and to what extent these different offending patterns are related to IPV perpetration. Moreover, building upon existing research, we include several measures of IPV perpetration, namely psychological IPV, any physical IPV, and severe physical IPV, as well as persistent IPV. Based on the theoretical framework and prior research discussed above, two hypotheses have been formulated: (a) It is expected that those with more serious and persistent patterns of general offending are more likely to report psychological, any physical, and severe physical IPV perpetration, and (b) it is hypothesized that those involved in more serious and persistent general offending are more likely to display persistence in psychological, any physical, and severe physical IPV perpetration.

## Method

### Sample

The study uses data from the Longitudinal Cohort component of the larger PHDCN study (Earls & Buka, 1997; Earls et al., 2002). The Longitudinal Cohort component followed seven age cohorts of children and young adults (aged 0, 3, 6, 9, 12, 15, and 18) over three waves, with approximately two years between waves.^1^ A stratified probability sampling approach was adopted to first randomly select neighborhoods clusters, then block groups within neighborhoods, and then potential participants. Both primary caregivers and respondents were interviewed for the younger cohorts, whereas only respondents were interviewed in the final (aged 18) cohort. Over 6,000 participants were interviewed across the different cohorts.

As the focus of this study is on general offending and IPV perpetration in young adulthood, we use data from the cohort of 18 year olds (*N* = 633, 50.2% males). In addition, as the aim of this study is to examine the relationship between developmental patterns in offending and IPV perpetration, a subsample was selected comprising respondents who participated in all three waves (*N* = 388).^2^ This subsample consists of 204 females (52.6%) and 184 (47.4%) males. With regard to race/ethnicity, the majority of the sample is either Hispanic (*n* = 146, 37.9%) or African American (*n* = 143, 37.1%). Another 77 respondents are Caucasian (20.0%), and 19 (5.0%) have other ethnic backgrounds. Four in five respondents (*n* = 310, 80.7%) were either in high school at the time of Wave 1, or had completed high school, while one in five had not completed high school. Almost 40% of young people lived in a medium SES (socioeconomic status) neighborhood, whereas about an equal proportion lived in low (29.6%) or high (31.2%) SES neighborhoods. Respondents’ average age in years at Wave 1 was 18.13 (*SD* = 0.34), 20.26 (*SD* = 0.63) at Wave 2, and 22.83 (*SD* = 0.59) at Wave 3 (Table 1).

**Table 1.** Demographic Characteristics of the Subsample (*N* = 388).

Demographic characteristics	*n*	%
Gender		
Male	184	47.4
Female	204	52.6
Ethnicity		
Hispanic	146	37.9
African American	143	37.1
White	77	20.0
Other	19	5.0
In/completed high school	310	80.7
Neighborhood SES		
Low	115	29.6
Medium	152	39.2
High	121	31.2
	*M*	*SD*
Age at Wave 1	18.13	0.34
Age at Wave 2	20.26	0.63
Age at Wave 3	22.83	0.59
	*n*	%
In a relationship in Wave 1	292	75.3
In a relationship in Wave 3	274	70.6
In a relationship in both waves	216	55.7

*Note.* SES = socioeconomic status.

For the analyses examining the association between patterns in general offending and IPV perpetration, subsamples of respondents who were in a relationship, or had been in the past year, were selected for subsequent analyses. This resulted in subsamples of 292 and 274 respondents for the analyses examining associations between general offending and IPV perpetration in Wave 1 and Wave 3, respectively, and 216 respondents for the analyses on general offending and persistence in IPV perpetration.^3^

### Data and Measures

#### General offending

Data on general offending were collected in all three waves, using a Self-Report of Delinquency questionnaire (Huizinga et al., 1991). The 26 items that were included in all three waves were included in this study. Of these items, 18 measured nonviolent offenses and eight items captured violent offending. The offenses measured ranged from relatively minor offenses such as driving without a license to serious offenses such as robbery. Respondents were asked if and how often they had engaged in the different delinquent acts in the past year. For this study, the dichotomous items were used to construct a variety scale for self-reported offending per wave, representing the number of different offenses each respondent had reported. A variety scale is preferred over both dichotomous measures, which minimize variation among respondents, and frequency scales, as these tend to be highly skewed due to high frequencies of relatively minor acts (Sweeten, 2012). Reliability of the self-reported offending scale was good in Wave 1 (Cronbach’s alpha = .77), but slightly lower in Wave 2 (Cronbach’s alpha = .69) and Wave 3 (Cronbach’s alpha = .61).

#### IPV perpetration

Self-reported IPV perpetration in the past year was measured using the Conflict Tactics Scale (CTS) in Wave 1 and using items from the Revised Conflict Tactics Scale (CTS2) in Wave 3 (Straus, 1979; Straus et al., 1996). Respondents were asked whether they were involved with or dating someone in the past year. If respondents had been in more than one relationship in the past year, they were asked to answer the questions on IPV about their most significant relationship. In Wave 1, the psychological aggression scale consisted of six items, and for Wave 3, four of the items from the psychological aggression scale were included. For example, items asked how often respondents had insulted or swore at their partner, or had threatened to hit or throw something at their partner. Using these items, a measure of *psychological IPV* was constructed for each wave, indicating whether respondents had reported at least one of these acts in the past year. The reliability of both psychological aggression scales was good (Cronbach’s alpha Wave 1 = .82; Wave 3 = .73).

In both waves, three similar items were used to measure minor physical violence, namely items that asked how often respondents had thrown something at their partner, had pushed or shoved, or had slapped their partner. Severe physical violence was measured using six items in Wave 1 and four items in Wave 3. Both waves included items asking how often respondents had kicked or hit their partner, or they had used a knife or gun. For each wave, the variable *any physical IPV* captured whether respondents had engaged in any of the violent behaviors at least once in the past year. Reliability of both physical aggression scales was good (Cronbach’s alpha Wave 1 = .83; Wave 3 = .75). In addition, separate variables for *severe physical IPV* were constructed per wave, indicating whether one or more of the severely violent acts had happened at least once in the past year. The severe physical IPV scale was reliable in Wave 1 (Cronbach’s alpha = .71) but did not perform as well in Wave 3 (Cronbach’s alpha = .54). Finally, for each type of IPV, dichotomous *persistence* variables were constructed, for which respondents who engaged in a particular type of IPV in both waves were coded as persisting. To illustrate, respondents who engaged in a form of psychological IPV in both Waves 1 and 3 were coded as persisting in psychological IPV perpetration.

### Analysis

Descriptive statistics and bivariate analyses were used to describe the prevalence of IPV perpetration across Waves 1 and 3. In addition, group-based trajectory modeling was used to examine the development of self-reported offending over the three waves. This technique clusters respondents together that show a similar developmental pattern in offending (Nagin, 1999, 2005). Offending trajectories were estimated in STATA. A zero-inflated Poisson model was fitted because the dependent measures used count variables representing the number of different offenses committed per wave. Models with different numbers of groups were estimated, and the best solution was determined using the Bayesian information criterion (BIC). The model identified which offending trajectory respondents belong to, and also estimated posterior probabilities that respondents belong to each of the trajectory groups (Nagin, 2005).

Results of the group-based trajectory modeling were used in a series of binary logistic regression analyses, aimed at investigating the relationship between patterns in the development of self-reported offending and different measures of IPV perpetration. Namely, an independent categorical variable representing the offending group to which respondents were assigned was included in the regression analyses. The following demographic characteristics were included in the analyses as control measures: dichotomous variables for *gender* (female = 0, male = 1) and respondents’ *educational achievement* (0 = not in high school/not completed high school, 1 = finished high school/in high school); categorical variables for *race/ethnicity* (Hispanic, African American, Caucasian/Other) and *neighborhood SES* (low, medium, high). The dichotomous dependent variables in the different models were *psychological, any physical*, and *severe physical IPV perpetration* in Wave 1 and in Wave 3, as well as *persistence* in these different types of IPV perpetration across the waves.

## Results

### General Offending in Young Adulthood

General offending was measured across all three waves. Results showed that a considerable proportion of young adults engaged in some offending in Wave 1 (71.0%) when respondents were on average 18 years old, and this number had decreased to just over half of the respondents (52.8%) by Wave 3, when they averaged 23 years old. Nonviolent offending was more common, although a sizable proportion of young adults also reported involvement in violent offending (Table 2).

**Table 2.** Self-Reported Offending Across the Waves (*N* = 388).

Offending variables	Wave 1		Wave 2		Wave 3	
	*n*	%	*N*	%	*n*	%
Total offending	274	71.0	248	64.1	204	52.8
Nonviolent offending	242	62.7	228	58.9	192	49.7
Violent offending	171	44.3	117	30.2	83	21.5
General offending trajectories	Group sizes		Proportion male		Posterior probabilities	
	*n*	%	*N*	%	*M*	*SD*
Group 1: Non-offenders	105	27.1	23	21.9	0.89	0.10
Group 2: Low-rate offenders	209	53.9	99	47.4	0.87	0.13
Group 3: High-rate offenders	74	19.1	62	83.8	0.90	0.13
	Wave 1		Wave 2		Wave 3	
	*M*	*SD*	*M*	*SD*	*M*	*SD*
Group 1: Non-offenders						
Number of different offenses	0.17	0.38	0.12	0.32	0.14	0.40
Number of different nonviolent offenses	0.11	0.32	0.11	0.31	0.13	0.40
Number of different violent offenses	0.06	0.23	0.01	0.10	0.01	0.10
Group 2: Low-rate offenders						
Number of different offenses	2.11	1.49	1.50	1.27	1.00	1.10
Number of different nonviolent offenses	1.41	1.25	1.13	0.98	0.78	0.86
Number of different violent offenses	0.70	0.85	0.37	0.67	0.22	0.54
Group 3: High-rate offenders						
Number of different offenses	5.92	2.92	4.64	2.52	3.23	1.89
Number of different nonviolent offenses	3.86	2.07	3.18	1.93	2.27	1.39
Number of different violent offenses	2.05	1.44	1.46	1.18	0.96	0.96

**Figure 1.**
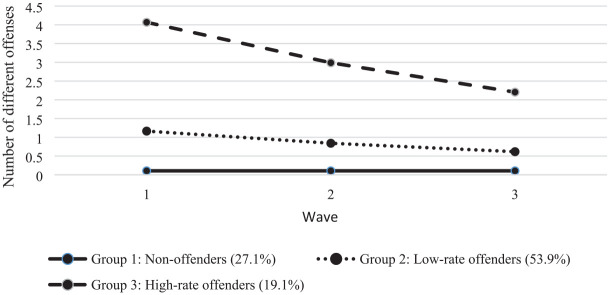
Three-group solution for self-reported general offending.

The results of group-based trajectory modeling indicated that there were three offending trajectories in the sample. Group 1, consisting of about 27%, was not, or only to a very limited extent, involved in offending across the three waves (for readability, we refer to this group as non-offenders). A large group of just over half of the sample was involved in some offending, and the number of different offenses they committed decreased over the waves (Group 2: low-rate offenders). About one fifth of the sample showed a higher rate of offending (Group 3: high-rate offenders). Although their offending behavior also decreased over the waves, their level of offending at Wave 3 was considerably higher compared with the other two groups (Figure 1, Table 2). Table 2 also shows that the average posterior probabilities per offending group are high, which means that respondents had a high probability of being assigned to the offending group that best resembled the development of their offending behavior across the waves (Nagin, 1999).

Table 2 also shows that, not surprisingly, the average number of different offenses committed is clearly highest in the high-rate offender group. Moreover, whereas the average number of different violent offenses reported is (very) low in the non-offender and low-rate offender groups, it is considerably higher in the high-rate offending group. Thus, the high-rate offending group shows a diverse pattern of both nonviolent and violent criminal behavior. Finally, even though many young adults engaged in some offending, the average number of offenses can be considered to be low, as the total offending scale measured 26 different types of offenses.

### IPV Perpetration in Young Adulthood

IPV perpetration was measured in Waves 1 and 3. Most respondents (91.4%) reported at least some form of psychological IPV perpetration in Wave 1 (Table 3). This proportion had decreased to just under 80% in Wave 3. Combining data from both waves showed that three quarters of the sample engaged in persistent psychological IPV.

**Table 3.** Prevalence of IPV Perpetration.

Total Sample	Wave 1 (*N* = 292)		Wave 3 (*N* = 274)		Waves 1 and 3 (*N* = 216)	
	*n*	%	*n*	%	*n*	%
Psychological IPV	267	91.4	214	78.1	166	76.9
Any physical IPV	138	47.3	76	27.7	45	20.8
Severe physical IPV	68	23.3	42	15.3	19	8.8
Males	(*n* = 132)		(*n* = 120)		(*n* = 90)	
Psychological IPV	117	88.6	90	75.0	65	72.2
Any physical IPV	36	27.3	24	20.0	11	12.2
Severe physical IPV	11	8.3	12	10.0	1	1.1
Females	(*n* = 160)		(*n* = 154)		(*n* = 126)	
Psychological IPV	150	93.8	124	80.5	101	80.2
Any physical IPV	102	63.8	52	33.8	34	27.0
Severe physical IPV	57	35.6	30	19.5	18	14.3

*Note.* IPV = intimate partner violence.

Furthermore, in Wave 1, almost half of the respondents reported physical IPV perpetration (47.3%). In Wave 3, the percentage of respondents perpetrating physical IPV had decreased to 27.7%. In addition, one in five respondents engaged in physical IPV perpetration in both waves. Although the vast majority of those reporting IPV perpetration engaged in relatively minor acts of physical IPV, 23.3% of young people reported committing at least one act of severe physical IPV perpetration in Wave 1, decreasing to 15.3% in Wave 3. Furthermore, 8.8% of young adults showed persistence in severe IPV perpetration.

### The Relationship Between General Offending and IPV Perpetration

Binary logistic regression analyses were used to examine the relationship between developmental patterns in self-reported offending and different measures of IPV perpetration, namely *psychological, any physical*, and *severe physical* IPV in both waves, and *persistence* in the different types of IPV perpetration across the waves. In all analyses, a categorical variable representing the offending group to which respondents belonged was included in the models, with the non-offender group serving as the reference category.

The results showed that in both Wave 1 (Table 4) and Wave 3 (Table 5), the two offender groups were significantly more likely to report engaging in psychological and (both any and severe) physical IPV perpetration, compared with the non-offender group. The effects were most pronounced for those involved in high-rate offending over the waves, indicating that those with the most serious offending pattern were at highest risk of engaging in IPV perpetration.

**Table 4.** Associations Between General Offending Trajectories and IPV Perpetration in Wave 1.

Variables	Psychological IPV		Any Physical IPV		Severe Physical IPV	
	*B*	*SE*	*B*	*SE*	*B*	*SE*
Male	−1.24*	0.51	−2.43***	0.36	−2.92***	0.52
African American	0.80	0.63	0.30	0.30	0.57	0.37
Caucasian/Other	−0.65	0.55	−0.18	0.40	−0.42	0.53
In/completed high school	0.24	0.58	−0.85*	0.35	−1.24**	0.39
Neighborhood SES medium	−1.36^†^	0.70	−0.11	0.33	−0.20	0.38
Neighborhood SES high	−0.98	0.75	−0.52	0.38	−0.36	0.46
Group 2: Low-rate offenders	1.13*	0.52	0.80*	0.34	1.23**	0.43
Group 3: High-rate offenders	2.19**	0.80	2.20***	0.50	2.63***	0.63
Constant	2.80***	0.77	0.82	0.43	−0.57	0.48
Nagelkerke *R*²	.17		.31		.33	

*Note.* IPV = intimate partner violence; SES = socioeconomic status.

† *p* < .10. *p < .05. **p < .01. ***p < .001.

**Table 5.** Associations Between General Offending Trajectories and IPV Perpetration in Wave 3.

Variables	Psychological IPV		Any Physical IPV		Severe Physical IPV	
	*B*	*SE*	*B*	*SE*	*B*	*SE*
Male	−1.10**	0.37	−1.20**	0.35	−1.14**	0.43
African American	0.98*	0.41	0.74*	0.34	0.36	0.41
Caucasian/Other	0.20	0.44	−0.04	0.46	−0.66	0.64
In/completed high school	−0.16	0.42	−0.46	0.36	−0.20	0.44
Neighborhood SES medium	0.51	0.40	−0.53	0.35	−0.57	0.42
Neighborhood SES high	−0.05	0.44	−0.46	0.41	−0.46	0.49
Group 2: Low-rate offenders	1.00**	0.38	1.14**	0.40	1.28*	0.54
Group 3: High-rate offenders	4.03***	1.09	1.85***	0.53	1.84**	0.67
Constant	0.46	0.46	−1.11*	0.47	−2.00**	0.61
Nagelkerke *R*²	.25		.18		.14	

*Note.* IPV = intimate partner violence; SES = socioeconomic status.

* *p* < .05. **p < .01. ***p < .001.

Furthermore, females were significantly more likely than males to report psychological and physical IPV perpetration in both waves. Those who were still in high school or had finished high school were less likely to report (any and severe) physical IPV perpetration in Wave 1, compared with those who had not completed high school. In addition, African Americans were significantly more likely to report psychological IPV and physical IPV in Wave 3 than Hispanics and Caucasians. Living in a medium SES neighborhood was associated with a reduced likelihood of psychological IPV perpetration in Wave 1, compared with coming from a low SES neighborhood, albeit the effect was marginally significant.

Moreover, those in the high-rate offending group were also significantly more likely to show *persistence* in psychological and (severe) physical IPV perpetration, compared with non-offenders (Table 6). Furthermore, low-rate offenders had a significantly higher likelihood of showing persistence in psychological IPV perpetration as well, and a marginally significantly increased risk of persistent severe physical IPV. Similar to the models that examined the relationship between offending patterns and IPV perpetration by wave, females were more likely than males to engage in persistent IPV, and African Americans were at increased risk of showing persistence in psychological IPV compared with Hispanics and Caucasians.

**Table 6.** Associations Between General Offending Trajectories and Persistence in IPV Perpetration.

Variables	Psychological IPV		Any Physical IPV		Severe Physical IPV	
	*B*	*SE*	*B*	*SE*	*B*	*SE*
Male	−1.11**	0.40	−1.38**	0.46	−3.27**	1.10
African American	1.08*	0.44	0.57	0.42	0.43	0.61
Caucasian/Other	0.53	0.48	0.14	0.55	−0.39	0.93
In/completed high school	−0.15	0.47	−0.45	0.43	0.20	0.71
Neighborhood SES medium	0.17	0.45	−0.09	0.42	−0.32	0.61
Neighborhood SES high	−0.29	0.48	−0.66	0.51	−0.37	0.70
Group 2: Low-rate offenders	0.90*	0.41	0.63	0.47	1.34^†^	0.81
Group 3: High-rate offenders	2.83***	0.75	1.48*	0.61	2.43*	0.98
Constant	0.51	0.51	−1.24*	0.57	−3.06**	1.00
Nagelkerke *R*²	.21		.14		.25	

*Note.* IPV = intimate partner violence. SES = socioeconomic status.

† *p* < .10. *p < .05. **p < .01. ***p < .001.

Taken together, the analyses demonstrated that involvement in general offending was associated with an increased likelihood of most forms of IPV perpetration across the waves, as well as with persistent IPV perpetration. The high-rate offender group in particular was significantly more likely to engage in (persistent) IPV perpetration, indicating that, although any offending is associated with an increased risk of IPV perpetration, it is especially those with a more diverse pattern of general offending who are at highest risk of perpetrating IPV.

## Discussion

This study used an urban and ethnically diverse sample of young adults to examine the relationship between patterns in the development of general offending and psychological and physical IPV perpetration in young adulthood. By doing so, this study extends the existing literature by illustrating longitudinally how general criminal careers relate to several measures of IPV perpetration, including psychological IPV and persistence in IPV perpetration.

With regard to self-reported general offending in young adulthood, the findings confirmed, as anticipated (e.g., Gottfredson & Hirschi, 1990; Sweeten et al., 2013), that offending behavior decreased during young adulthood, from a prevalence rate of 71% in Wave 1 to 53% in Wave 3. However, group-based trajectory modeling revealed different developmental patterns, namely a group of respondents who did not, or only to a very limited extent, engage in offending; a group of low-rate offenders who showed some offending across the waves, and for whom the number of offenses reported declined with age; and a group of respondents who displayed a high rate of offending, although they also showed a decrease in offending over the waves.

Furthermore, the study showed that a considerable proportion of young adults engaged in IPV perpetration. In particular, psychological IPV perpetration was common in the sample, although it too decreased from 91% in Wave 1 to 78% in Wave 3. These rates are similar to findings from other self-report studies among young adults. For example, Magdol et al. (1997) found that 90% and Kim et al. (2008) found that 72% of young adults reported psychological IPV perpetration. In addition, almost half (47%) of the respondents in this study perpetrated physical IPV in Wave 1, compared with 28% in Wave 3. These findings are also comparable to existing self-report studies, although results vary. For instance, Novak and Furman (2016) found a prevalence rate of physical IPV of 51%, while Magdol et al. (1997) and Kim et al. (2008) found that 29% and 28% of young adults reported physical IPV perpetration, respectively. Furthermore, over three quarters of the sample engaged in psychological IPV across both waves, and about one in five respondents reported persistent physical IPV perpetration.

The regression analyses indicated that compared with non-offenders, those involved in general offending, and especially those showing a persistent and diverse offending pattern, were significantly more likely to report psychological, any physical, and severe physical IPV perpetration, thereby confirming the first hypothesis. Moreover, and in line with the second hypothesis, the high-rate offending group was also at greater risk of showing persistence in psychological and (severe) physical IPV, whereas the low-rate offending group was only significantly associated with persistence in psychological IPV, and marginally significantly related to persistence in severe physical IPV. Thus, general offending was associated with an increased likelihood of IPV perpetration, and those with a more persistent and diverse offending pattern were particularly at risk of (persistent) IPV perpetration.

These findings are consistent with general theories of crime and violence, which—although not always explicitly stated—expect a relationship between different forms of antisocial and violent behavior, such as between general offending and IPV perpetration, due to a shared underlying etiology (Fagan & Wexler, 1987; Felson & Lane, 2010; Gottfredson & Hirschi, 1990; Moffitt, 1993). Compared with non-offenders, those involved in any offending had a significantly increased likelihood of perpetrating IPV, pointing to an important overlap between general crime and IPV perpetration. Moreover, findings of this study add to prior research in this area by demonstrating that those with a more diverse offending pattern throughout young adulthood were at increased risk of not only physical IPV but psychological IPV perpetration too, as well as persistent IPV perpetration (e.g., Herrenkohl et al., 2007; Piquero et al., 2014). This pattern of comorbidity suggests that it is helpful to view IPV perpetration as part of a broader pattern of antisocial and criminal behavior. Importantly, even though rates of IPV perpetration in this sample decreased with age—a finding similar to other longitudinal self-report studies on situational couple violence (W. L. Johnson et al., 2015; Kim et al., 2008; Shortt et al., 2012)—the results indicated that those with the highest rates and diversity of offending are at particularly high risk of developing a persistent pattern of IPV perpetration that may extend beyond young adulthood.

Besides the strong connection between general and IPV offending, some demographic characteristics were also related to IPV perpetration. For example, a high school education was a protective factor for physical IPV perpetration in Wave 1. In addition, in some, but not all, models, African Americans were at increased risk of IPV perpetration compared with Hispanics, which is largely in line with prior research (e.g., Capaldi et al., 2012). For instance, Caetano et al. (2005) found that African Americans and Hispanics were more likely to report physical IPV compared with Caucasians, and that persistence in IPV over a 5-year period was more common in African American couples. However, it appears that the association between race/ethnicity and IPV is likely partly explained by neighborhood socioeconomic disadvantage (Benson et al., 2004).

In the sample under study, more females than males reported engaging in the different types of IPV perpetration, except for psychological IPV perpetration in both waves (results not shown). This difference was expected, as prior survey research on young adults and newlywed couples has found that rates of IPV perpetration among females are often similar to males (Ehrensaft et al., 2004; W. L. Johnson et al., 2015; Magdol et al., 1997; Novak & Furman, 2016; O’Leary et al., 1989; Schumacher & Leonard, 2005). However, there are several measurement issues to note with regard to potential gender symmetry in IPV perpetration.

First, while IPV is often measured using a version of the Conflict Tactics Scale (CTS or CTS2; Straus, 1979; Straus et al., 1996), a few scholars have questioned whether this instrument can accurately measure IPV perpetration (R. P. Dobash et al., 1992). Most notably, they argue that the CTS inadequately captures the contexts of and motivations for violent incidents, power dynamics in the relationship, and consequences of IPV (R. P. Dobash & Dobash, 2004; R. P. Dobash et al., 1992). Some scholars have found that when compared with violence perpetrated by males, violence perpetrated by females is often reactive, is less likely to cause an injury, to be perceived as threatening, and to generate fear (Archer, 2000; Hester, 2013; Miller & White, 2003; Whitaker et al., 2007). These gendered nuances are arguably not well captured using a quantitative instrument like the CTS. It is also worth noting that we have not distinguished between heterosexual and same-sex relationships, and nor were we able to examine those respondents with other gender identities.

Related to the above-mentioned measurement issues, M. P. Johnson (2008) argued that surveys of general population samples are likely to capture one type of IPV perpetration, namely situational couple violence, which is relatively minor, mutual violence between partners. In this study, significant overlap was found between IPV perpetration and victimization (results not shown), suggesting that the type of IPV measured in the study indeed most likely resembles situational couple violence, rather than intimate terrorism. This later expression of violence is characterized by male-perpetrated serious, persistent violence and controlling behavior, usually observed in shelter or criminal justice samples. Moreover, while the CTS includes a psychological aggression scale, the items used to measure this type of IPV do not capture coercive controlling behavior such as demanding, threatening, and surveillance behaviors, all of which are features of intimate terrorism (Dutton & Goodman, 2005; M. P. Johnson, 2008; Stark, 2007). Future longitudinal research in this area could address this shortcoming by including an instrument measuring coercive control. This would enable researchers to examine the development of various forms of IPV including control tactics alongside general criminal behavior over time (Dutton et al., 2006; Lehmann et al., 2012).

Furthermore, in this study, the original version of the CTS was used to measure IPV in Wave 1, while items from the revised CTS were used in Wave 3. The separate items were used to construct scales for psychological and physical IPV in both waves, but some of the items were not completely comparable across the waves. Moreover, IPV was not measured in Wave 2, which is unfortunate, as it would have given more insight into the developmental patterns in IPV perpetration. Future research including multiple longitudinal measurements throughout adolescence and young adulthood of both general offending as well as the various aspects of IPV perpetration would help to further examine the development of IPV perpetration, and processes of persistence and desistance, in relation to the development of general criminal behavior. In addition, examining criminal and IPV development in relation to other individual, relationship, and neighborhood factors would be particularly useful to further our understanding of IPV persistence and desistance (Capaldi & Kim, 2007; Cattaneo & Goodman, 2005; Emery et al., 2011; Jain et al., 2010; Walker et al., 2013).

Finally, the findings of this study need to be understood in the context of the sample under study, which consisted of urban young adults from a range of different socioeconomic and ethnic backgrounds. Although the stratified probability sampling approach used in the PHDCN study is a strength in terms of achieving a representative sample of young adults from Chicago, it is unclear to what extent findings generalize to other contexts, including other (nonurban) geographical areas and other countries. The United States differs from most other industrialized nations, for example, with regard to rates of concentrated poverty and socioeconomic inequality, welfare provisions, penal climate, and access to weapons. Therefore, future research using different samples, especially from other Western, non–English speaking countries, would shed more light on the generalizability of the findings on the relationship between general crime and IPV.

Although not without limitations, this study adds to the existing body of literature that connects criminal career research and the study of IPV development. Therefore, the findings provide important avenues for prevention and intervention. Similar to other self-report studies, the findings demonstrate that IPV perpetration in young adulthood is common, pointing to the importance of general prevention aimed at educating young people about healthy relationships. Moreover, the results of this study indicate that intervention efforts should focus on young offenders in general, as they are particularly at risk of engaging in IPV perpetration. Moreover, a small group displaying a persistent and diverse pattern of both nonviolent and violent offending were particularly at risk of perpetrating psychological and (severe) physical IPV across time. This conclusion is in line with the notion that a small group of offenders are responsible for a large proportion of crimes and harm, and therefore that it would be useful to focus resources and intervention efforts on this group of “power few” or persistent, high-risk offenders (Moffitt et al., 2002; Robinson & Clancy, 2020; Sherman, 2007).

Focusing prevention efforts on high-risk general offenders is especially important as IPV, especially the more minor incidents, often does not become known to the authorities. Therefore, it is important for those working with youths involved with criminal justice agencies to recognize that these individuals are at higher risk of becoming involved in IPV as well. Intervening early in the criminal careers of young offenders, especially the small group of more serious general offenders, is likely a crucial step to preventing them from developing persistent patterns of abusive, antisocial, and criminal behavior that will likely extend into adulthood and even into later life (Verbruggen et al., 2019). Moreover, preventing persistent IPV is also particularly important, as research has indicated that among those who engage in IPV, a small proportion of perpetrators show a pattern of repeated and severe IPV, and are responsible for a large part of the harm associated with this IPV, as well as for a large proportion of incidents and police callouts (Bland & Ariel, 2015; Sherman et al., 2016). In conclusion, our research findings reinforce previous calls for developing a more holistic strategy of intervening with young offenders to reduce IPV (Klein, 2015). Our study adds further evidence that for such a strategy to be effective, it must be premised on an understanding that the entire cohort is at heightened risk of becoming, if they are not already, involved in IPV perpetration.

## Notes

1. Data collection for Wave 1 took place between 1994 and 1997, between 1997 and 2000 for Wave 2, and between 2000 and 2002 for Wave 3.

2. Respondents who participated in all three waves (*N* = 388) were not significantly different from those who did not, in terms of their age at Wave 1, ethnicity, neighborhood socioeconomic status (SES), or involvement in criminal behavior across the waves. However, those who participated in all three waves were better educated at Wave 1, as there was a relatively larger proportion of young adults who were still in or had completed high school, compared with those who were no longer in high school, χ²(1, 624) = 17.48, *p* < .001. Moreover, more females than males were included in the subsample, although this difference was marginally significant, χ²(1, 633) = 3.18, *p* = .075.

3. Those who were in a relationship at Wave 1 (*n* = 292) did not differ significantly from those who were not in a relationship in Wave 1 (*n* = 96) with regard to gender, ethnicity, level of education, neighborhood SES, or age at Wave 1. However, those in a relationship were significantly more likely to report involvement in criminal behavior at Wave 1, χ²(1, 386) = 16.88, *p* < .001. To illustrate, 81.4% of those who reported offending at Wave 1 were in a relationship in Wave 1, compared with 61.6% of those who were not involved in offending. Similarly, those who were in a relationship at Wave 3 (*n* = 274) did not differ significantly from those who were not in a relationship in Wave 3 (*n* = 114) with regard to ethnicity, educational level, neighborhood SES, age at Wave 3, or involvement in offending at Wave 3. However, more females than males were in a relationship at Wave 3, χ²(1, 388) = 4.92, *p* < .05.
